# A TCN-BiLSTM and ANR-IEKF Hybrid Framework for Sustained Vehicle Positioning During GNSS Outages

**DOI:** 10.3390/s26010152

**Published:** 2025-12-25

**Authors:** Senhao Niu, Jie Li, Chenjun Hu, Junlong Li, Debiao Zhang, Kaiqiang Feng

**Affiliations:** 1 National Key Laboratory of Dynamic Testing Technology for Extreme Environment Optoelectronics, North University of China, Taiyuan 030051, China; niuhh1022@163.com (S.N.); hcj74839@gmail.com (C.H.); lijunl07@nuc.edu.cn (J.L.); fkq0809@163.com (K.F.); 2 School of Electronic Information Engineering, Taiyuan University of Science and Technology, Taiyuan 030024, China; zhangdebiao@tyust.edu.cn

**Keywords:** Global Navigation Satellite System (GNSS), Inertial Navigation System (INS), GNSS/INS integrated navigation system, Iterated Extended Kalman Filter (IEKF), temporal convolutional network (TCN), Bidirectional LSTM (BiLSTM), GNSS outage

## Abstract

**Highlights:**

**What are the main findings?**

- An Adaptive Noise-Regulated Iterated Extended Kalman Filter (ANR-IEKF) has been developed as a robust estimation kernel that dynamically adjusts noise characteristics in real time. This approach effectively addresses the uncertainties associated with deep learning predictions while ensuring numerical stability.
- An integration of the Temporal Convolutional Network and Bidirectional Long Short-Term Memory (TCN-BiLSTM) is developed to leverage the TCN’s capability to extract local temporal features from INS data while utilizing BiLSTM’s ability to model long-range, bidirectional trajectory dependencies.
- A hybrid ANR-IEKF + TCN-BiLSTM architecture is proposed. Within this framework, the TCN-BiLSTM generates precise pseudo-GNSS measurements from raw INS data, which are then adaptively fused by the ANR-IEKF.

**What are the implications of the main finding?**

- The hybrid TCN-BiLSTM architecture establishes a transformative framework for compensating positioning errors by decoding complex nonlinear relationships between raw INS data and vehicle displacement. This method consistently outperforms traditional models, thereby enabling reliable navigation in environments with degraded GNSS signals.
- Comprehensive real-vehicle experiments conducted across various driving scenarios demonstrate that the integrated ANR-IEKF+TCN-BiLSTM algorithm exhibits superior accuracy and robustness during GNSS outages. This finding establishes it as a reliable solution to the inherent limitations of conventional GNSS/INS systems.

**Abstract:**

The performance of integrated Global Navigation Satellite System and Inertial Navigation System (GNSS/INS) navigation often declines in complex urban environments due to frequent GNSS signal blockages. This poses a significant challenge for autonomous driving applications that require continuous and reliable positioning. To address this limitation, this paper presents a novel hybrid framework that combines a deep learning architecture with an adaptive Kalman Filter. At the core of this framework is a Temporal Convolutional Network and Bidirectional Long Short-Term Memory (TCN-BiLSTM) model, which generates accurate pseudo-GNSS measurements from raw INS data during GNSS outages. These measurements are then fused with the INS data stream using an Adaptive Noise-Regulated Iterated Extended Kalman Filter (ANR-IEKF), which enhances robustness by dynamically estimating and adjusting the process and observation noise statistics in real time. The proposed ANR-IEKF + TCN-BiLSTM framework was validated using a real-world vehicle dataset that encompasses both straight-line and turning scenarios. The results demonstrate its superior performance in positioning accuracy and robustness compared to several baseline models, thereby confirming its effectiveness as a reliable solution for maintaining high-precision navigation in GNSS-denied environments. Validated in 70 s GNSS outage environments, our approach enhances positioning accuracy by over 50% against strong deep learning baselines with errors reduced to roughly 3.4 m.

## 1. Introduction

GNSS (Global Navigation Satellite System) and INS (Inertial Navigation System) are both essential technologies in navigation, yet each has inherent limitations [1,2,3]. GNSS is vulnerable to signal outages in complex environments, such as urban canyons, while INS experiences accumulating errors due to drift in its integration process, resulting in a decline in navigation accuracy over time. Nevertheless, these two systems are highly complementary. The long-term stability of GNSS in open-sky conditions effectively constrains the error growth of INS. Conversely, the high autonomy and independence from external signals of INS can mitigate the gaps during GNSS outages caused by environmental factors. As a result, GNSS/INS integrated navigation systems have been widely adopted in applications such as autonomous driving, aerial vehicle navigation, and marine surveying, owing to their high precision, robust performance, and broad applicability [4,5,6,7].

The Kalman Filter (KF) is a widely utilized methodology for data fusion. However, the standard KF is limited to linear systems, making it inadequate for the inherently nonlinear integration of GNSS and INS [8,9]. To tackle the challenges of nonlinear system estimation, various enhanced KF frameworks and their derivatives have been developed. The Extended Kalman Filter (EKF) addresses nonlinearity by linearizing the system model around the current state estimate, which has made it a prevalent tool in GNSS/INS navigation [10,11]. However, its propensity to disregard higher-order terms during linearization can lead to substantial errors in highly nonlinear contexts. In contrast, the Unscented Kalman Filter (UKF) utilizes a deterministic sampling method, employing sigma points to approximate the mean and covariance of the state distribution via an unscented transform. This approach frequently yields greater accuracy than the EKF. Despite its enhanced capability in managing nonlinearities, the UKF is computationally more demanding than the EKF, presenting a significant limitation for real-time applications [12,13].

In environments characterized by high real-time demands and moderate nonlinearity, the Iterated Extended Kalman Filter (IEKF) has been introduced. The IEKF improves upon the standard EKF in accuracy by performing multiple iterations to re-linearize the nonlinear measurement function around the updated state estimate, thereby refining the solution [14]. Furthermore, by carefully controlling the number of iterations, the computational cost of the IEKF can be kept between that of the EKF and the UKF, making it a suitable choice for applications that require both timeliness and enhanced accuracy [15]. Subsequent research has sought to further enhance the IEKF. For instance, Junbo Zhao et al. [16] proposed a robust IEKF based on the generalized maximum likelihood approach (GM-IEKF), which demonstrated greater reliability than the EKF. Similarly, Tao Wen et al. [17] developed an IEKF augmented by the Least Squares Under-measurement (LSU) technique. This modified algorithm captures more higher-order information than the traditional EKF and has shown applicability in train positioning and tracking systems. Therefore, selecting an appropriate variant of the Kalman Filter is crucial for improving the reliability and precision of GNSS/INS integrated navigation systems.

Advancements in data fusion algorithms can enhance the reliability and robustness of GNSS/INS systems; however, these improvements fundamentally depend on the availability of measurement data. A complete absence of GNSS signals, which frequently occurs in challenging environments, renders these fusion algorithms ineffective. During extended GNSS outages, the system reverts to a stand-alone INS, leading to a rapid deterioration in navigation accuracy due to the unbounded growth of inertial sensor errors. This inherent limitation highlights the critical importance of maintaining positioning accuracy following a GNSS outage. Addressing this issue remains a significant challenge in the field of integrated navigation [18,19,20].

To address the degradation of positioning accuracy in GNSS/INS integrated systems during GNSS outages, various solutions have been proposed. A notable category of these methods involves the integration of supplementary sensors. For example, Jing Dong et al. [21] developed a vision-aided INS/odometry integrated system, which exhibited a significant improvement in accuracy compared to a stand-alone INS. Similarly, Guojian He et al. [22] proposed a GNSS/LiDAR fusion framework that utilizes the complementarity between GNSS positioning and LiDAR-SLAM to achieve high-precision pose estimation and map construction in GNSS-denied environments. Furthermore, Junbing Cheng et al. [23] introduced a GNSS/INS/Vision framework that employs a multi-constraint dynamic feature point elimination method to enhance positioning accuracy during GNSS signal interruptions. These methods effectively mitigate the divergence of positioning errors and enhance accuracy by fusing data from alternative sensors in the absence of GNSS signals. However, the integration of additional sensors inevitably increases the system’s hardware costs, power consumption, and structural complexity.

Another category of solutions utilizes neural networks to develop models that generate pseudo-GNSS measurements, which are subsequently integrated with INS data to mitigate the degradation in positioning accuracy. For instance, Ying Xu et al. [24] proposed a motion-constrained GNSS/INS integration method based on a Backpropagation Neural Network (MC-BP). Haowen Wang et al. [25] introduced an Online Semi-Supervised Transformer (OSS-Transformer) model designed to function effectively during GNSS outages while maintaining low computational overhead. Similarly, Yidi Chen et al. [26] presented a Long Short-Term Memory (LSTM)-assisted GNSS/INS system that leverages IMU error characteristics to suppress the error divergence of INS in the absence of GNSS signals. Zhengsong Wang et al. [6] proposed an enhanced vehicle self-localization method based on a hybrid Convolutional Neural Network (CNN)-LSTM architecture. Wulong Dai et al. [27] demonstrated that a CNN-BiLSTM-based error compensation method can enhance positioning accuracy during GNSS interruptions. These networks have shown considerable effectiveness in correcting navigation errors across various applications. In terms of managing temporal navigation data, models such as Gated Recurrent Unit (GRU), LSTM, and Transformer are particularly well suited. The GRU model is generally more effective for short-term sequences, while the Transformer architecture excels with very long-term sequences [28]. However, the LSTM model often retains an advantage for general long-sequence tasks. Therefore, selecting an appropriate neural network model tailored to the specific application scenario is essential for the accurate prediction of pseudo-GNSS measurements [26,29,30].

The predictive performance of a neural network model is significantly influenced by the quality and informational content of its raw input data. When the input data are contaminated by substantial noise or interference, the model’s generalization capability may be severely impaired, limiting its ability to discern underlying patterns from the training data. Consequently, to optimize navigation prediction accuracy, the data fusion algorithm must be appropriately aligned with the neural network model. Recent research supports this assertion. For example, Xiaoliang Meng et al. [31] developed an Improved Robust Adaptive Kalman Filter (IRAKF) that enhances prediction accuracy by estimating the measurement noise covariance of pseudo-GNSS information. Similarly, Jiageng Liu et al. [32] combined an improved IEKF with a multiple Long Short-Term Memory (multi-LSTM) network to enhance vehicle positioning accuracy during GPS outages. In another study, Xiaokai Wei et al. [33] proposed an adaptive Kalman Filter based on the maximum versoria criterion (MVC) and integrated it with a bidirectional gated recurrent unit (BiGRU), which improved the accuracy and robustness of data fusion, thereby providing more effective information samples for neural network training. Collectively, these studies illustrate that a well-aligned integration of data fusion algorithms and neural network models can significantly enhance the accuracy of information prediction.

In GNSS/INS-integrated navigation applications, the state estimation problem can be framed as a time-series prediction task characterized by a feature distribution that dynamically evolves with motion patterns and environmental conditions [34]. This framework renders temporal sequence models as a suitable option for predicting pseudo-measurements during GNSS outages. Nonetheless, the practical effectiveness of this approach is hindered by several critical challenges. First, the input data typically derive from high-noise, low-cost Micro-Electro-Mechanical Systems (MEMS) inertial sensors. The inherent instability of these sensors introduces significant errors into the data fusion process, thereby providing the neural network with imprecise and noisy input samples. Second, the computational demands of advanced data fusion algorithms can be considerable, potentially compromising the real-time performance and efficiency of the navigation system. Finally, in long-sequence prediction tasks, standard network architectures may inadequately capture the complex temporal dependencies present in the data, resulting in suboptimal training performance and diminished prediction accuracy. Collectively, these factors restrict the effectiveness and broader applicability of neural networks in practical navigation systems [35,36,37].

To tackle the challenges outlined above, this paper presents a seamless navigation measurement strategy aimed at improving positioning and prediction accuracy. The proposed strategy incorporates an IEKF optimized with an Adaptive Noise Regulation (ANR) mechanism to ensure robust data fusion and filtering. Additionally, it employs a hybrid model that combines a Temporal Convolutional Network (TCN) with a Bidirectional Long Short-Term Memory network (BiLSTM) to enhance prediction precision. The primary contributions of this work are summarized as follows:

(1) Introduction of an Adaptive Noise-Regulated IEKF (ANR-IEKF): This component aims to enhance the robustness and accuracy of data fusion in complex scenarios, thereby providing superior filtering performance.
(2) Development of a Hybrid TCN-BiLSTM Network: A novel neural network architecture is proposed to generate accurate pseudo-GNSS measurements from inertial data by effectively capturing spatio-temporal features.
(3) Construction and Validation of a Unified Framework: The integrated ANR-IEKF and TCN-BiLSTM framework undergoes extensive evaluation on real-world datasets. The results demonstrate its superior performance compared to state-of-the-art benchmarks.

## 2. Materials and Methods

**Figure 1:**
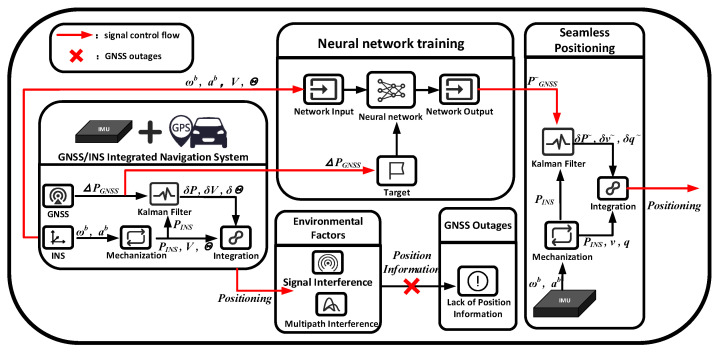
Overall operational block diagram of the algorithm and network model.

This section outlines the core algorithms and models utilized in this study, focusing on the data fusion algorithm and the neural network model designed for the GNSS/INS integrated navigation system. The overall workflow of the proposed system is illustrated in Figure 1.

This flowchart outlines the systematic procedure of the proposed method. In practice, raw inertial measurement data from the IMU serve as the input. These data are processed by the TCN-BiLSTM network to produce intermediate pseudo-GNSS position estimates. Subsequently, these estimates are fused with the INS mechanization output within the ANR-IEKF framework to generate the final, corrected navigation states (position, velocity, and attitude). This end-to-end sequence from sensory input to refined output fully defines the reproducible data flow.

### 2.1. Filtering Algorithm

Before analyzing the IEKF, LM-IEKF, and ANR-IEKF algorithms, it is crucial to introduce the state vector. This study employs a 15-dimensional GNSS/INS integrated navigation error model as the state vector within the data fusion algorithm [38]. The definition of this error state vector is presented as follows:

(1) $$x={\left[{\left(\delta r^{n}\right)}^{T},{\left(\delta v^{n}\right)}^{T},\phi^{T},\nabla^{T},\epsilon^{T}\right]}^{T}$$

where $\delta r^{n}$ and $\delta v^{n}$ represent the position error and velocity error in the navigation frame, respectively; ϕ denotes the attitude error vector. The vectors ∇ and ϵ represent the constant biases of the accelerometer and gyroscope, respectively. All components, namely $\delta r^{n}$, $\delta v^{n}$, ϕ, ∇ and ϵ, are three-dimensional vectors. The state equation and observation equation of the nonlinear system model in the EKF are as follows:

(2) $$x_{k}=f(x_{k-1},u_{k-1})+w_{k-1},\;\;\;w_{k-1}\sim N(0,Q_{k-1})$$

(3) $$z_{k}=h(x_{k})+m_{k},\;\;\;m_{k}\sim N(0,R_{k})$$

In the expressions above, $x_{k}$ denotes the error state vector at the *k*-th epoch. The function f(⋅) represents the nonlinear state transition function. The vector $w_{k}$ is the process noise. The control input vector $u_{k-1}$ in the state prediction equation corresponds to the INS specific force and angular rate measurements. The vector $z_{k}$ is the observation vector, which is defined as the difference between the GNSS-derived position and velocity solutions and the corresponding predicted values from the INS. The function h(⋅) is the nonlinear observation function. The vector $m_{k}$ represents the observation noise, which accounts for the noise and errors present in the GNSS observations. In this paper, both $w_{k}$ and $m_{k}$ are considered zero-mean Gaussian white noise. The matrices $Q_{k}$ and $R_{k}$ are the process noise covariance matrix and the observation noise covariance matrix at time *k*, respectively.

#### 2.1.1. IEKF

The traditional EKF linearizes nonlinear models by applying a first-order Taylor expansion around the current state estimate. This method may produce considerable linearization errors in complex environments, leading to diminished estimation accuracy or potential filter divergence [39,40]. In contrast, the IEKF executes multiple iterations within a single time step. During each iteration, relinearization occurs around a new and more precise state estimate, significantly mitigating the errors introduced by a single linearization step. The prediction step of the IEKF mirrors that of the EKF:

(4) $${\hat{x}}_{k|k-1}=f({\hat{x}}_{k-1|k-1},u_{k-1})$$

(5) $$P_{k|k-1}=F_{k-1}P_{k-1|k-1}F_{k-1}^{T}+Q_{k-1}\;,\;F_{k-1}=\frac{\partial f}{\partial X}{|}_{{\hat{X}}_{k-1|k-1},u_{k-1}}$$

where ${\hat{x}}_{k|k-1}$ is the prior state estimate at time *k* derived from the posterior state estimate ${\hat{x}}_{k-1|k-1}$ at time *k*−1 and the control input vector $u_{k}$ through the function f(⋅). The matrix $P_{k|k-1}$ is the prior estimate error covariance matrix. The matrix $F_{k-1}$ is the state transition matrix, which is evaluated at point ${\hat{x}}_{k-1}$.The IEKF algorithm begins by initializing the prior state estimate and setting the iteration index *i* = 0:

(6) $${\hat{x}}_{k}^{0}={\hat{x}}_{k|k-1},\;\;\;i=0$$

After initializing ${\hat{x}}_{k}$, the iterative process begins from iteration *i* = 1. For the *i*-th iteration, the observation matrix $H_{k}^{i}$, the observation vector $z_{k}^{\left(i\right)}$, and the Kalman gain $K_{k}^{\left(i\right)}$ are computed. Subsequently, the state vector and the covariance matrix are updated using this information:

(7) $$H_{k}^{i}={\left.\frac{\partial h}{\partial x}\right|}_{{\hat{x}}_{k}^{i}}$$

(8) $$r_{k}^{i}=z_{k}-h({\hat{x}}_{k}^{i})$$

(9) $$K_{k}^{i}=P_{k|k-1}{\left(H_{k}^{i}\right)}^{T}{\left(H_{k}^{i}P_{k|k-1}{\left(H_{k}^{i}\right)}^{T}+R_{k}\right)}^{-1}$$

(10) $${\hat{x}}_{k}^{i+1}={\hat{x}}_{k}^{i}+K_{k}^{i}r_{k}^{i}$$

(11) $$P_{k}^{i+1}=(I-K_{k}^{i}H_{k}^{i})P_{k|k-1}$$

Upon completion of each iteration, the state vector is evaluated against the following criterion:

(12) $$\|{\hat{x}}_{k}^{i+1}-{\hat{x}}_{k}^{i}\|<\eta \;\mathrm{or}\;i\geq N_{\max}$$

The iterative process terminates when the difference between two consecutive error state vectors is less than a predefined convergence threshold η or when the number of iterations reaches a specified maximum $N_{\max}$. Upon termination, the final iterated error state vector $x_{k}^{N}$ and the updated covariance matrix $P_{k}^{N}$ are output:

(13) $${\hat{x}}_{k|k}={\hat{x}}_{k}^{N},\;\;\;P_{k|k}=P_{k}^{N}$$

#### 2.1.2. LM-IEKF

The IEKF mitigates the linearization error inherent in the standard EKF, which results from linearizing at a single operating point, by iteratively refining the linearization point to enhance estimation accuracy. However, the fixed iterative step size can result in convergence to local minima or even divergence, particularly when the initial state estimate is significantly inaccurate or when the system exhibits pronounced nonlinear behavior. To address this limitation, the Levenberg–Marquardt Iterative Extended Kalman Filter (LM-IEKF) integrates an adaptive damping mechanism based on the Levenberg–Marquardt optimization algorithm. This mechanism dynamically adjusts the damping factor, facilitating a smooth transition between the Gauss–Newton method and the gradient descent strategy, thereby enhancing both convergence performance and overall robustness. [39,41] The prediction step of the LM-IEKF aligns with that of the IEKF, and its initialization procedure is defined as follows:

(14) $${\hat{x}}_{k}^{0}={\hat{x}}_{k|k-1},\;\;\;\lambda^{0}=\lambda_{0},\;\;\;i=0$$

where $\lambda_{0}$ is the initial damping factor. During the computation of the Kalman gain $K_{k}^{i}$, the LM-IEKF introduces a damping term $\lambda^{i}I$ into the observation noise covariance matrix. The modified equation is expressed as follows:

(15) $$K_{k}^{i}=P_{k|k-1}{\left(H_{k}^{i}\right)}^{T}{\left(H_{k}^{i}P_{k|k-1}{\left(H_{k}^{i}\right)}^{T}+{\tilde{R}}_{k}^{i}\right)}^{-1}$$

(16) $${\tilde{R}}_{k}^{i}=R_{k}+\lambda^{i}I$$

where I denotes the 15 × 15 identity matrix, while matrix ${\tilde{R}}_{k}^{i}$ represents the observation noise covariance matrix incorporating the damping term $\lambda^{i}I$, which equips the conventional IEKF with an adaptive iterative step size capability. The adjustment rule for the damping factor $\lambda^{i}$ is defined as follows:

(17) $$\lambda^{i+1}=\left\{\begin{array}{ll} \max\left(\frac{\lambda^{i}}{\alpha },\lambda_{\min}\right) & \;\rho >\rho th\\ \min\left(\lambda^{i}\cdot \alpha ,\lambda_{\max}\right) & \rho \leq \rho th \end{array}\right.$$

where $\lambda_{\min}$ is the minimum damping factor, which prevents numerical instability caused by an excessively small λ. $\lambda_{\max}$ is the maximum damping factor, which prevents the step size from becoming too small due to an overly large λ. The parameter α is the damping adjustment coefficient, controlling the magnitude of changes to λ. The gain ratio ρ is compared against a gain ratio threshold ρth to determine whether the iteration has reached an effective critical value. When ρ>ρth, the iteration is considered effective, and the step size can be increased by reducing λ. Conversely, if ρ<ρth, the iteration is ineffective, and the step size should be decreased by increasing λ. The expression for ρ is given as follows:

(18) $$\rho =\frac{\|r_{k}^{i}{\|}^{2}-\|z_{k}-h({\hat{x}}_{k}^{i+1}){\|}^{2}}{{\left(K_{k}^{i}r_{k}^{i}\right)}^{T}(\lambda^{i}K_{k}^{i}r_{k}^{i}+{\left(H_{k}^{i}\right)}^{T}R_{k}^{-1}r_{k}^{i})}$$

The gain ratio ρ is defined as the ratio of the actual reduction in error to the predicted reduction in error from the model. This ratio effectively quantifies the validity of each iteration. The modifications described above constitute the key improvements of the LM-IEKF over the standard IEKF. By incorporating the damping term, the LM-IEKF ensures the effectiveness of the iterative process. This enhancement not only preserves the advantages of the IEKF but also improves the robustness and convergence performance of the algorithm.

#### 2.1.3. ANR-IEKF

The introduction of the damping term $\lambda^{i}I$ in the LM-IEKF, while ensuring iterative validity, acts uniformly on every dimension of the observation noise covariance matrix $R_{k}$. That is,

(19) $${\tilde{R}}_{k}^{i}=\left[\begin{array}{cc} {R^{i}}_{\mathrm{GNSSp}}+\lambda^{i}I_{3} & 0_{3\times 3}\\ 0_{3\times 3} & {R^{i}}_{\mathrm{GNSSv}}+\lambda^{i}I_{3} \end{array}\right]$$

where ${R^{i}}_{\mathrm{GNSSp}}$ and ${R^{i}}_{\mathrm{GNSSv}}$ represent the position and velocity components of the GNSS observation noise matrix, respectively. This isotropic adjustment mechanism imposes uniform noise inflation across all observation dimensions when an anomaly occurs in any single dimension. Consequently, an outlier in one dimension adversely affects all observational dimensions.

To address this limitation, the ANR-IEKF reinterprets the damping factor as a physically meaningful time-varying noise regulation term $\Lambda_{k}^{i}$. The computation of the Kalman gain $K_{k}^{\left(i\right)}$ is accordingly modified as follows:

(20) $$K_{k}^{i}=P_{k|k-1}{\left(H_{k}^{i}\right)}^{T}{\left(H_{k}^{i}P_{k|k-1}{\left(H_{k}^{i}\right)}^{T}+{{\tilde{R}}^{\prime }}_{k}^{i}\right)}^{-1}$$

(21) $${{\tilde{R}}^{\prime }}_{k}^{i}=R_{k}+\Lambda_{k}^{i}$$

The time-varying noise regulation term $\Lambda_{k}^{i}$ is defined as

(22) $$\Lambda_{k}^{i}=\mathrm{diag}\left(\sigma_{1,k}^{i},\sigma_{2,k}^{i},\ldots ,\sigma_{m,k}^{i}\right)$$

(23) $$\sigma_{j,k}^{i}=\beta \cdot |r_{j,k}^{i}|+\gamma \cdot \sigma_{j,k}^{i-1}\;\;for\;j=1,2,\ldots ,m$$

where $\sigma_{j,k}^{i}$ represents an adaptively estimated standard deviation for the time-varying measurement noise affecting the j-th measurement component at time k and iteration i, *m* is the dimension of the measurement vector, β denotes the mapping gain from residuals to noise, and γ is the forgetting factor for historical noise. This formulation indicates that the current noise estimate is composed of the contribution from the current residual and the memory of historical noise, thereby considering both instantaneous data quality and maintaining temporal continuity.

The forgetting factor γ, which governs the weight of historical noise estimates, must satisfy 0<γ<1 to guarantee boundedness. In alignment with the short-term correlation characteristics typical of navigation errors, γ is set to 0.8. This configuration effectively smooths out transient anomalies while maintaining sufficient responsiveness to track actual noise variations. The noise mapping gain β scales the dimensionless residual into a physical noise standard deviation. Based on an analysis of the residual magnitude in the training data, β is set to 0.5, implying that a one-unit residual induces an additional 0.5 units of noise estimation. This configuration ensures that the regulation intensity is commensurate with the actual observation uncertainty.

When a specific observation dimension exhibits an abnormally large residual, denoted as *r^i^_j,k_* ≫ 0, the ANR-IEKF produces the following outcome:

(24) $$r_{j,k}^{i}\gg 0\to \sigma_{j,k}^{i}\gg 0\to K_{j,k}^{i}\approx 0$$

As evidenced by the expression above, an abnormally large residual causes a significant increase in the time-varying noise, which in turn drives the Kalman gain $K_{j,k}^{i}$ asymptotically toward zero. This mechanism effectively suppresses the aberrant observation, thereby constituting an efficient automatic outlier isolation and detection strategy.

The modifications outlined above represent significant enhancements of the ANR-IEKF compared to the LM-IEKF. By integrating a time-varying noise regulation term, the ANR-IEKF overcomes the LM-IEKF’s limitations in managing outliers. This method converts the mathematical damping factor into a physical noise estimate, allowing for independent noise estimation across each dimension. Additionally, it features an automatic mechanism for outlier isolation and detection, which substantially maintains the accuracy of the data fusion algorithm.

### 2.2. The Prediction Model for the GNSS/INS Integrated Navigation System

First, it is essential to select suitable inputs and outputs for constructing the prediction model, ensuring its applicability in GNSS/INS integrated navigation systems during GNSS signal outages while also enhancing the training efficiency and accuracy of the model. Our primary objective is to predict the system state based on the available INS information when GNSS data are not accessible. The inertial coordinate system is defined as the East–North–Up (E-N-U) frame for system modeling. Let $\Delta P_{GNSS}$ represent the position change vector derived from the GNSS between two consecutive time steps in the navigation coordinate system, which is defined as follows:

(25) $$\Delta P_{GNSS}=\left[\begin{array}{c} \Delta x_{\mathrm{GNSS}}\\ \Delta y_{\mathrm{GNSS}}\\ \Delta z_{\mathrm{GNSS}} \end{array}\right]=\iint \left[C_{b}^{n}a_{ib}^{b}(t)-\left(2\omega_{ie}^{n}+\omega_{en}^{n}\right)\times V^{n}(t)+g^{n}\right]dtdt$$

This expression reveals the relationship between GNSS position changes and raw INS measurements where, $\Delta x_{\mathrm{GNSS}}$, $\Delta y_{\mathrm{GNSS}}$, and $\Delta z_{\mathrm{GNSS}}$ represent the position changes along the x, y and z axes, respectively. $C_{b}^{n}$ is the rotation matrix from the body frame to the navigation frame. $a_{ib}^{b}$ denotes the acceleration measured in the body frame. $V^{n}(t)$ represents the vehicle’s velocity in the navigation frame. $g^{n}$ refers to the local gravity vector expressed in the navigation frame. $\omega_{ie}^{n}$ and $\omega_{en}^{n}$ denote the Earth’s rotation rate and the transport rate projected in the navigation frame, respectively. The expression for $\omega_{en}^{n}$ is given as follows:

(26) $$\omega_{en}^{n}=\left[\begin{array}{c} \frac{V_{N}}{R_{M}+h}\\ -\frac{V_{E}}{R_{N}+h}\\ -\frac{V_{E}\tan L}{R_{N}+h} \end{array}\right]$$

where $V_{N}$ and $V_{E}$ represent the north and east velocity components of the vehicle in the navigation frame, respectively. R_M_ and R_N_ denote the meridian radius of curvature and the prime vertical radius of curvature of the Earth’s ellipsoid, respectively, which are functions of latitude, L denotes the latitude of the vehicle, and h indicates the altitude relative to the reference ellipsoid. From Equation (26), it can be observed that the position change is primarily influenced by the velocity $V^{n}$ in the navigation frame. In the error-state model used for filtering, the cross-coupling terms associated with $\omega_{ie}^{n}$ and $g^{n}$ in the state transition matrix are negligible for the short update intervals and moderate dynamics considered in this study, and they are therefore omitted to simplify the filter implementation. The expression for $C_{b}^{n}$ and its time derivative are given as follows [27]:

(27) $$C_{b}^{n}=\left[\begin{array}{ccc} \cos\theta \cos\psi & \cos\theta \sin\psi & -\sin\theta \\ \sin\phi \sin\theta \cos\psi -\cos\phi \sin\psi & \sin\phi \sin\theta \sin\psi +\cos\phi \cos\psi & \sin\phi \cos\theta \\ \cos\phi \sin\theta \cos\psi +\sin\phi \sin\psi & \cos\phi \sin\theta \sin\psi -\sin\phi \cos\psi & \cos\phi \cos\theta \end{array}\right]$$

(28) $$\frac{d}{dt}C_{b}^{n}=C_{b}^{n}(\omega_{nb}^{b}\times )=C_{b}^{n}(\omega_{ib}^{b}\times )-(\omega_{in}^{n}\times )C_{b}^{n}$$

where ϕ, θ, and ψ represent the roll, pitch, and yaw angles, respectively. These can be denoted as shown below:

(29) $$\Theta ≜\left[\begin{array}{c} \phi \\ \theta \\ \psi \end{array}\right]$$

where Θ represents the attitude angles. As discussed above, $C_{b}^{n}$ is primarily influenced by Θ and $\omega_{ib}^{b}$. In summary, $\Delta P_{GNSS}$ is closely related to $a_{ib}^{b}$, $\omega_{ib}^{b}$, $V^{n}$, and Θ. Therefore, the input–output model of the GNSS/INS integrated navigation system can be expressed as shown below:

(30) $$\left\{\begin{array}{l} {Model}_{Input}:\;\left[a_{ib}^{b}\;\;\omega_{ib}^{b}\;\;V^{n}\;\;\Theta \right]\\ {Model}_{Output}:\;\left[\Delta P_{GNSS}\right]=\left[\Delta x_{GNSS}\;\;\Delta y_{GNSS}\;\;\Delta z_{GNSS}\right] \end{array}\right.$$

The neural network model within this integrated navigation system operates in two distinct phases: training and prediction. When GNSS signals are available, the system enters the training phase. During this phase, the network is trained to map the INS-derived parameters $a_{ib}^{b}$, $\omega_{ib}^{b}$, $V^{n}$ and Θ to the corresponding target values $\Delta P_{GNSS}$. When GNSS signals are unavailable, the system switches to the prediction phase. The INS-derived parameters $a_{ib}^{b}$, $\omega_{ib}^{b}$, $V^{n}$ and Θ are fed into the pre-trained network, which then generates predicted values to compensate for the missing GNSS information. These predictions are subsequently fused with the INS data using the ANR-IEKF algorithm.

Having established the input and target for the neural network model, the next step is to design a network architecture that can effectively extract relevant features and provide accurate predictions.

### 2.3. TCN-BiLSTM

The BiLSTM network is a recurrent neural model that effectively utilizes both past and future information to understand the current context. This architecture exhibits remarkable capability in predicting time-series data, making it well suited for generating pseudo-GNSS measurements [42,43]. While its gating mechanism mitigates the vanishing gradient problem during training, the model’s capacity to capture pertinent information from long sequences is still constrained. Important features in extended sequences may become diluted or intertwined with irrelevant noise across multiple time steps, potentially undermining the accuracy of the pseudo-GNSS signals.

To overcome this limitation, we utilize a temporal convolutional network (TCN) as a front-end feature extractor for the bidirectional long short-term memory (BiLSTM) network. The dilated convolutional operations of the TCN are especially effective in capturing long-range dependencies in sequential data [44,45]. The proposed TCN-BiLSTM model consists of four essential components: a data preprocessing layer, a TCN layer, a BiLSTM layer, and a navigation decoding layer.

**Figure 2:**
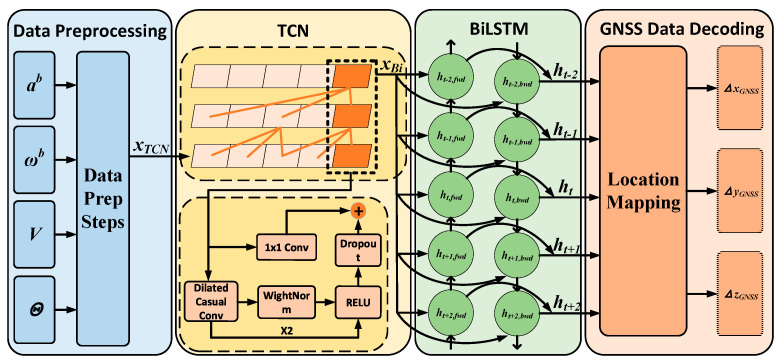
TCN-BiLSTM network framework.

While recent advances, particularly Transformer-based architectures, have demonstrated superior capability in modeling very long-range dependencies through self-attention mechanisms, their high computational complexity and substantial data requirements may limit deployment in resource-constrained real-time navigation systems. In contrast, the proposed TCN-BiLSTM hybrid architecture seeks a more practical balance: the TCN provides efficient long-range feature extraction with linear complexity, and the BiLSTM offers precise local contextual modeling. This design prioritizes operational efficiency and robustness for pseudo-GNSS signal generation without relying on extensive pre-training or excessive compute resources. The overall workflow of the model is depicted in Figure 2.

Data Preprocessing Layer: To mitigate potential issues such as training difficulties, slow convergence, and suboptimal performance when raw INS data are directly fed into the TCN model, a preprocessing step is applied. This step involves calculating the sliding-window mean ${\bar{x}}_{k}\in R^{12}$ of the raw data sequence $x_{i}\in R^{12}$, where L denotes the number of samples in the sliding window. The calculation is defined as follows:

(31) $$x_{i}={Model}_{Input}\;,\;{\bar{x}}_{k}=\frac{1}{L}\sum_{i=1}^{N}x_{i}$$

where *N* denote the size of the sliding time window (number of samples) used for averaging. 

Subsequently, the computed mean values are subjected to zero-bias removal and gravity alignment procedures. These steps aim to minimize the discrepancy between the pseudo-GNSS sequences generated by the neural network during GNSS-denied periods and the corresponding actual trajectory:

(32) $${\overset{⌣}{x}}_{i}=R_{k}\left(x_{i}-{\bar{x}}_{k}\right)-g_{0},\;\;\;g_{0}={\left[\begin{array}{lll} 0 & 0 & g \end{array}\right]}^{⊤}$$

where ${\overset{⌣}{x}}_{i}$ denotes the preprocessed input after zero-bias removal and gravity alignment, $R_{k}$ represents the direction cosine matrix used for gravity alignment, and $g_{0}={\left[\begin{array}{lll} 0 & 0 & g \end{array}\right]}^{⊤}$ is the local gravity vector in the navigation frame, with g being the gravitational acceleration constant, while ${\overset{⌣}{x}}_{i}$ is normalized as follows:

(33) $${\tilde{x}}_{i}={\left(\Sigma_{k}+\varepsilon I\right)}^{-1}{\overset{⌣}{x}}_{i},\;\;\;\Sigma_{k}=\mathrm{diag}\left(\sigma_{k}^{\left(1\right)},\ldots ,\sigma_{k}^{\left(12\right)}\right)$$

where $\sigma_{k}^{\left(j\right)}$ is the *j*-dimensional sample standard deviation, $\Sigma_{k}\in R^{12\times 12}$ is the diagonal matrix formed by the sample deviations, and ε is a regularization factor included to suppress numerical errors and gradient explosion when the sensor output approaches a constant value. After normalization, the input to the TCN layer, denoted as $X_{TCNInput}$, is obtained as follows:

(34) $$X_{TCNInput}={\left[\begin{array}{lll} {\tilde{x}}_{k-L+1} & \cdots & {\tilde{x}}_{k} \end{array}\right]}^{⊤}$$

**Figure 3:**
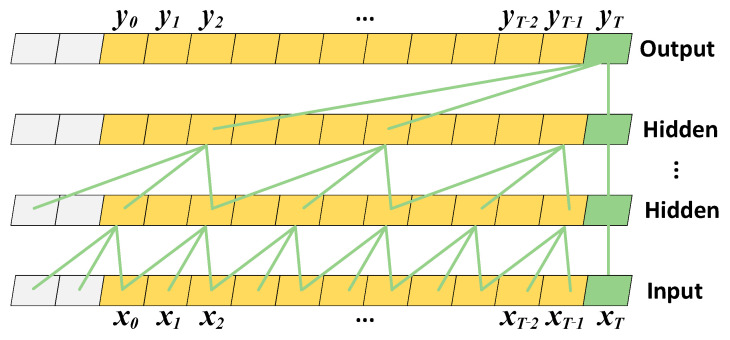
TCN network framework.

TCN Layer: The architecture of the TCN is depicted in Figure 3. By utilizing the strided sampling feature of dilated convolutions, this model allows each layer to encompass a wider receptive field of the input sequence without significantly increasing the number of parameters.

Simultaneously, the TCN can extract high-level features from INS data in parallel, providing high-resolution temporal data for the subsequent BiLSTM network and mitigating gradient decay in long-sequence learning. The output of the TCN layer, denoted as $Y_{TCNOutput}\in R^{C_{\mathrm{out}}}$, is expressed as follows:

(35) $$Y_{TCNOutput}=ReLU\left(W_{\mathrm{res}}X_{\mathrm{TCNInput}}^{\left(t\right)}+\sum_{j=0}^{K-1}W[j]X_{\mathrm{TCNInput}}^{\left(t-\mathrm{dj}\right)}+b\right)\;,\;W[j]\in R^{C_{\mathrm{out}\;}\times C_{\mathrm{in}\;}}$$

(36) $$C_{\mathrm{out}}=\dim(Y_{TCNOutput}),\;\;\;C_{\mathrm{in}}=\dim(X_{\mathrm{TCNInput}}^{\left(t\right)})$$

where $W_{\mathrm{res}}\in R^{C_{\mathrm{out}}\times C_{in}}$ is the residual mapping matrix, which is employed to alleviate gradient degradation. The parameter *K* represents the kernel size, which determines the span of original samples covered by a single convolution operation. The matrix $W[j]\in R^{C_{\mathrm{out}}\times C_{in}}$ denotes the *j*-th weight matrix of the causal dilated convolution kernel. This causal dilated convolution slides along the temporal dimension, effectively capturing local dynamic features within the sequence. The vector $b\in R^{C_{\mathrm{out}}}$ is the bias term. The dilation factor *d* enables the efficient capture of long-range dependencies in the time series by expanding the receptive field of the convolution kernel without increasing computational complexity. The local features extracted by the TCN significantly enhance the signal-to-noise ratio, allowing the BiLSTM to focus on long-range contextual modeling. This cooperative mechanism effectively suppresses error drift and accelerates convergence.

Furthermore, the dilated convolutional structure of the TCN provides a transparent mapping between input perturbations and output variations. Unlike recurrent networks where temporal dependencies are entangled in hidden states, the fixed receptive field and sparse connectivity of the TCN allow the contribution of each input step to be analytically bounded and traced, offering a principled foundation for understanding its temporal reasoning without post hoc explanatory methods.

**Figure 4:**
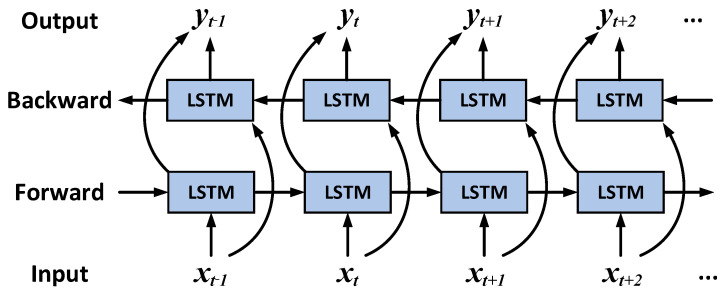
BiLSTM network framework.

BiLSTM Layer: The architecture of the BiLSTM is illustrated in Figure 4. While the TCN effectively extracts local features via its dilated convolutional and causal constraint mechanisms, its unidirectional modeling inherently restricts its capacity to leverage future contextual information. Additionally, it faces representational bottlenecks when addressing complex, non-stationary long-range dependencies.

The bidirectional recurrent architecture of the BiLSTM facilitates the simultaneous integration of historical and future information, thereby enhancing the model’s capacity for comprehensive contextual understanding. This effectively addresses the limitations of the TCN in global semantic integration and dynamic dependency modeling. The prediction process of the BiLSTM is as follows:

(37) $$X_{BiInput}=Y_{TCNOutput}$$

(38) $$h_{t,fwd},c_{t,fwd}=LSTM_{fwd}\left(X_{BiInput}^{\left(t\right)},h_{t-1,fwd},c_{t-1,fwd};\theta_{fwd}\right)$$

(39) $$h_{t,bwd},c_{t,bwd}=LSTM_{bwd}\left(X_{BiInput}^{\left(t\right)},h_{t-1,bwd},c_{t-1,bwd};\theta_{bwd}\right)$$

where $h_{t,fwd}\in R^{H}$ and $h_{t,bwd}\in R^{H}$ represent the forward and backward hidden states, respectively, while $c_{t,fwd}\in R^{H}$ and $c_{t,bwd}\in R^{H}$ denote the corresponding forward and backward cell states. The parameter *H* determines the network capacity by defining the number of hidden units. The matrices $\theta_{fwd}$ and vectors $\theta_{bwd}$ are the trainable weights and bias parameters.

After obtaining the bidirectional outputs $h_{t,fwd}$ and $h_{t,bwd}$ at each time step *t*, they are concatenated and fused through a hidden state integration operation:

(40) $$h_{t}=\left[h_{t,\;fwd\;};h_{t,\;bwd\;}\right]\in R^{2H}$$

Upon completion of both forward and backward processing, the BiLSTM generates its final output as follows:

(41) $$Y_{BiOutput}=\left[h_{1},\ldots ,h_{T}\right]\in R^{T\times 2H}$$

where $Y_{BiOutput}$ denotes the output of the BiLSTM network, and *T* represents the total number of time steps. The steps described above constitute the prediction process of the BiLSTM.

Navigation Decoding Layer: Following the output of high-dimensional temporal features from the BiLSTM, the navigation decoding layer translates these features into pseudo-GNSS measurement signals. This process facilitates a semantic transformation from abstract features to positional variations, allowing the generated signals to be directly employed in the data fusion algorithm, thus effectively mitigating positioning errors.

## 3. Simulation Verification and Experimental Design

This section outlines the simulation setup and experimental design specifically tailored to evaluate and compare the data fusion performance of the proposed ANR-IEKF with that of the LM-IEKF. The experiments aim to quantitatively assess the relative advantages of the ANR-IEKF, particularly regarding its accuracy, robustness, and capacity to manage anomalous observations.

### 3.1. Simulation Verification

**Figure 5:**
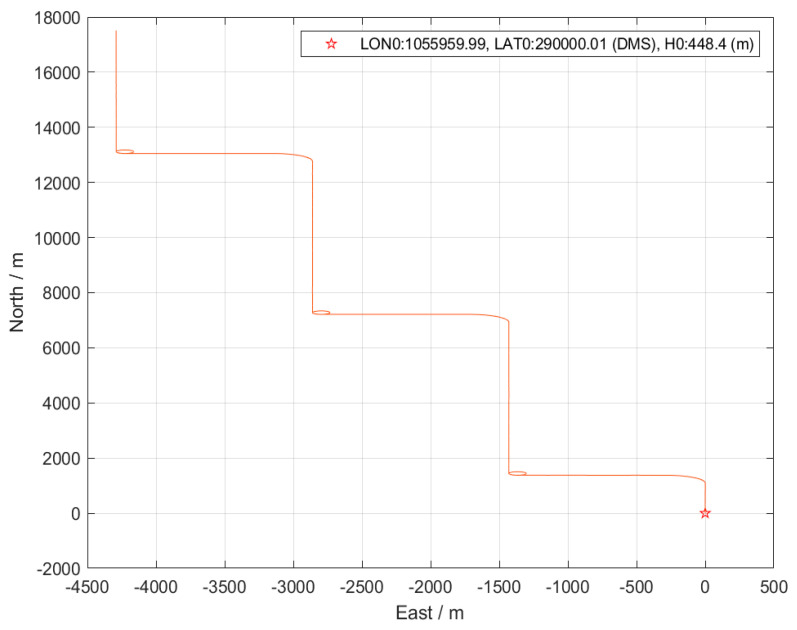
Vehicle trajectory from the dataset.

A comparative analysis was performed using a real-world dataset to assess the performance of the proposed ANR-IEKF relative to the LM-IEKF under nominal GNSS availability. This dataset comprises raw measurements from an MEMS-based INS and a GNSS receiver. The fundamental parameters, including the initial state vector and the process noise covariance matrix, were configured identically for both filters to facilitate a fair comparison. Figure 5 illustrates the vehicle trajectory derived from the dataset employed in this study.

**Figure 6:**
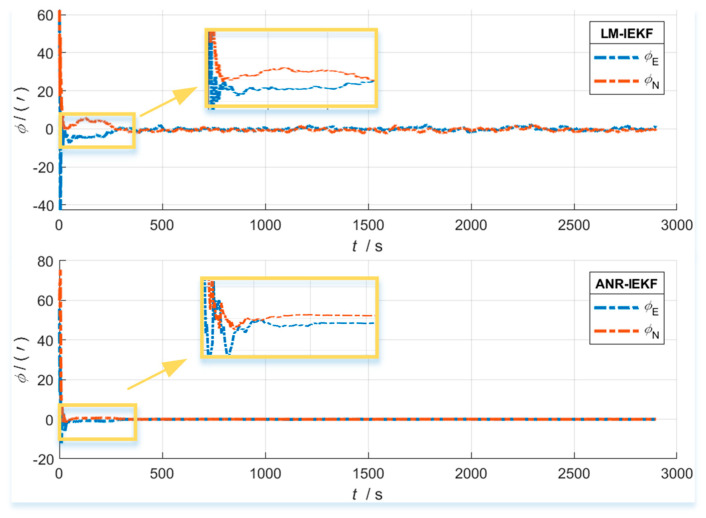
Attitude error comparison between LM-IEKF and ANR-IEKF.

**Figure 7:**
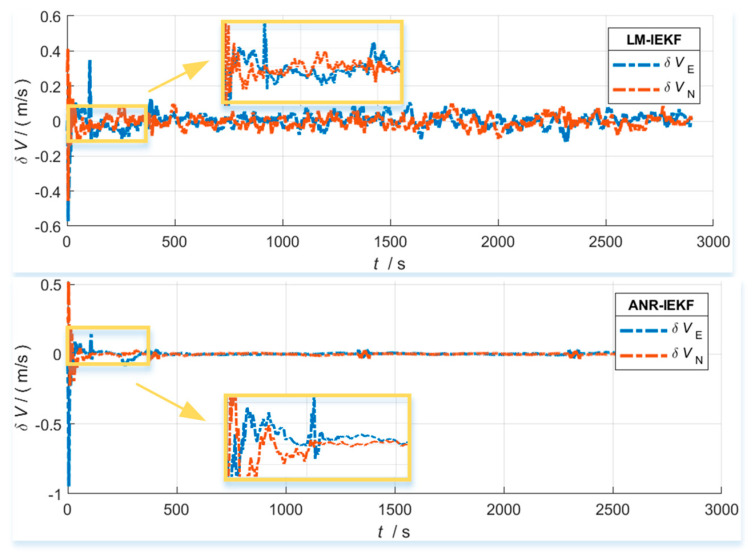
Velocity error comparison between LM-IEKF and ANR-IEKF.

**Figure 8:**
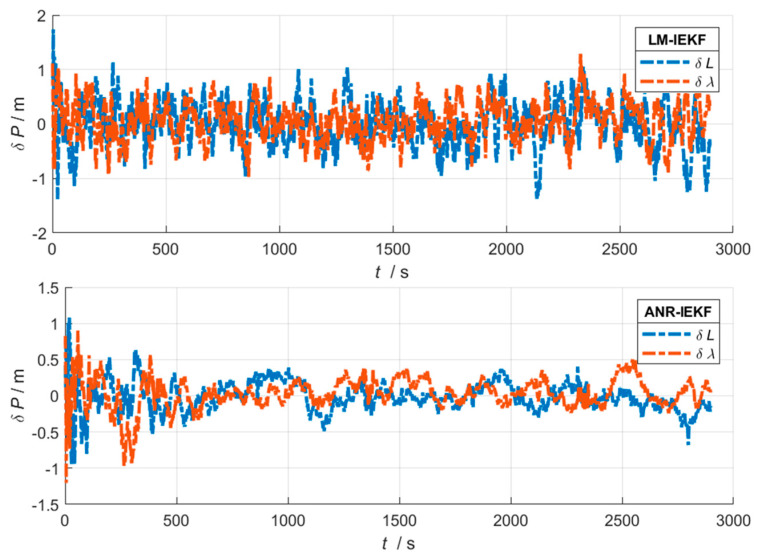
Position error comparison between LM-IEKF and ANR-IEKF.

Figure 6, Figure 7 and Figure 8 present a comparative analysis of the performance of the LM-IEKF and the proposed ANR-IEKF algorithms, highlighting their estimation errors in attitude, velocity, and position. Overall, the ANR-IEKF exhibits markedly enhanced accuracy and stability across all states.

In comparing attitude angle errors, both filters demonstrate satisfactory convergence, although their dynamic characteristics vary. During the initial convergence phase, the LM-IEKF exhibits a faster convergence rate with a smaller initial overshoot, allowing its error curve to enter the steady-state region more swiftly. Conversely, during extended steady-state operation, the ANR-IEKF provides enhanced stability, as indicated by diminished fluctuations in its error curve. This observation implies that while the LM-IEKF may excel in short-term transient response, the adaptive mechanism of the ANR-IEKF improves long-term estimation smoothness and robustness.

This enhancement in smoothness and robustness is directly attributable to the adaptive stability conferred by the Λ term. While the LM-IEKF’s fixed noise model leads to the persistent error oscillations visible in its steady state, the ANR-IEKF dynamically suppresses these oscillations through real-time noise regulation. This results in a tighter error bound and demonstrates the proposed filter’s robust convergence behavior under implicitly varying conditions.

The comparison of velocity error results indicates that the ANR-IEKF algorithm significantly surpasses the LM-IEKF regarding estimation accuracy and stability. Specifically, while the LM-IEKF demonstrates marked fluctuations in its error curves, the east and north velocity errors of the ANR-IEKF exhibit not only smaller magnitudes but also a close alignment with the zero-error baseline. This behavior reflects a high degree of smoothness and stability, thereby underscoring the essential role of the adaptive noise regulation mechanism in effectively mitigating error fluctuations and improving the reliability of velocity estimation.

The comparison of position errors clearly highlights the performance disparity between the two algorithms. As illustrated in the figure, the position error curve of the LM-IEKF not only displays significant fluctuations but also deviates markedly from the zero-error baseline, indicating considerable inaccuracies and instability in its positioning results. In contrast, the error curve of the ANR-IEKF remains effectively constrained within a narrow range close to zero and exhibits remarkable smoothness. This outcome provides compelling evidence that the adaptive mechanism of the ANR-IEKF leads to a substantial enhancement in the accuracy of position estimation, which is the primary objective of the navigation system, thereby underscoring its exceptional robustness and reliability.

In summary, the proposed ANR-IEKF algorithm consistently exhibits superior overall performance, particularly in maintaining estimation stability and accuracy across critical navigation states.

### 3.2. Experimental Equipment

**Figure 9:**
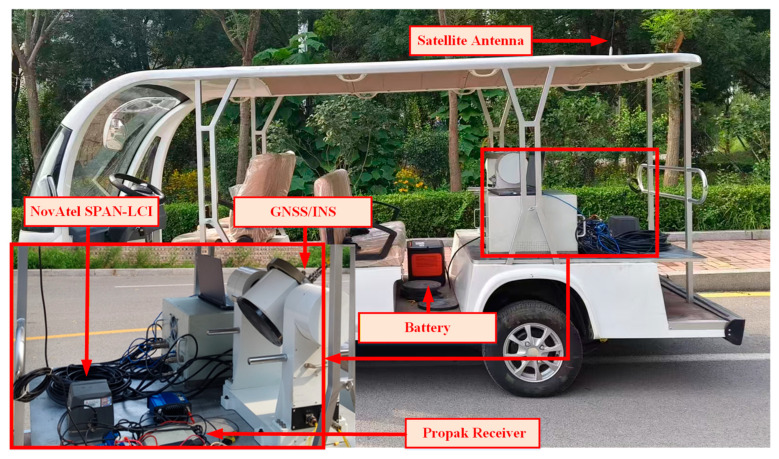
Vehicle experimental platform.

The vehicle-mounted experimental platform utilized for land vehicle tests is depicted in Figure 9. This platform features a GNSS/INS integrated navigation system, which includes a triaxial MEMS gyroscope, a triaxial accelerometer, and a GPS receiver, which are all designed for experimental data collection. The reference for quantitative comparison is provided by the tactical-grade, high-precision integrated navigation system, the NovAtel SPAN-LCI, along with its antenna. The INS sampling frequency is configured at 100 Hz, whereas the GPS receiver and the proposed system, as illustrated in the figure, operate at a frequency of 2 Hz.

The specifications and key parameters of the equipment are listed in Table 1.

Based on the equipment configuration and parameters presented in Figure 9, a corresponding experimental implementation plan was devised, and field tests were conducted at North University of China in Taiyuan, Shanxi Province. All algorithms utilized in this experiment were executed on an Intel Core i9-13900HX processor with 32 GB of RAM (Intel, Santa Clara, CA, USA). The core hyperparameters of the proposed TCN-BiLSTM model are summarized in Table 2.

### 3.3. Experimental Design

**Figure 10:**
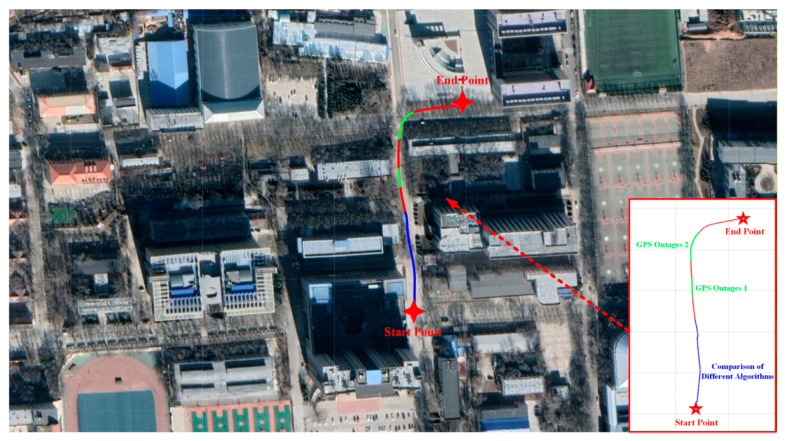
Real-world vehicle trajectory for algorithm evaluation.

To assess the effectiveness of the ANR-IEKF data fusion algorithm in real-world road tests and to evaluate the predictive performance of the ANR-IEKF+TCN-BiLSTM framework for generating pseudo-GNSS measurements, a comprehensive experimental campaign was designed and executed. Field tests were conducted on the campus of North University of China in Taiyuan, Shanxi Province. The vehicle trajectory recorded during these experiments is depicted in Figure 10.

The entire experimental trajectory extended approximately 1.3 km and lasted about 600 s, which was divided into two distinct segments. The first segment encompassed the initial 600 m of the vehicle’s path, during which various data fusion algorithms were evaluated in repeated trials to assess the effectiveness of the ANR-IEKF. This segment lasted roughly 210 s with the corresponding trajectory depicted by the blue segment in Figure 10. The second segment commenced at the 600 m mark and continued to the conclusion of the 1.3 km trajectory. In this segment, GNSS signal outages were manually simulated by deactivating the GNSS receiver for durations of 30 s and 70 s, respectively. The sections representing GNSS signal loss are highlighted in green in Figure 8. Throughout the experiments, the GNSS/INS integrated navigation system functioned in a loosely coupled configuration. The test environment presented multiple complex disturbances, including road bumps, surface wetness in certain areas, and varying vehicle dynamics such as turning and vertical oscillations. To reduce uncertainties associated with individual experiments, the proposed experimental protocol was repeated five times under identical configurations and along similar vehicle trajectories.

Specifically, the GPS Outages 2 segment was intentionally situated within a densely wooded area along the trajectory. This environment naturally induces GNSS signal attenuation and multipath effects, simulating a real-world challenging scenario beyond mere signal denial. The combination of complete outage simulation and this realistic signal-degraded environment provides a comprehensive test for the robustness of navigation algorithms under complex, mixed conditions.

## 4. Experimental Results and Analysis

### 4.1. Experimental Results from the First Trajectory Segment

**Figure 11:**
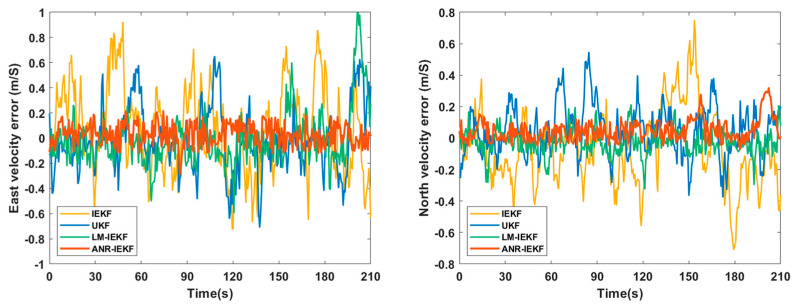
Comparison of east and north velocity errors for different filtering algorithms.

To assess the fundamental positioning performance of the ANR-IEKF algorithm under optimal signal conditions, this experimental phase conducted a comparative analysis between the ANR-IEKF and three conventional data fusion algorithms: the IEKF, LM-IEKF, and UKF. The experiment took place in a real-world vehicular scenario along the blue trajectory segment shown in Figure 10, lasting approximately 210 s, during which GNSS signals were continuously available. Figure 11 depicts the east and north velocity errors of the various fusion algorithms during vehicle operation, while Figure 10 presents the corresponding east and north position errors. The root mean square errors (RMSEs) for each algorithm are quantitatively summarized in Table 2.

Figure 11 illustrates the comparison of east and north velocity errors, highlighting the advantages of the ANR-IEKF algorithm in terms of estimation accuracy and stability. During the 210 s experiment, the IEKF displayed the most significant error fluctuations, revealing its vulnerability to model nonlinearities and noise. Although both the UKF and LM-IEKF exhibited lower error magnitudes than the IEKF, their outputs continued to show persistent oscillations throughout the experiment. In contrast, the ANR-IEKF consistently kept errors within a minimal range, demonstrating enhanced estimation consistency and robustness during the entire experimental period.

**Figure 12:**
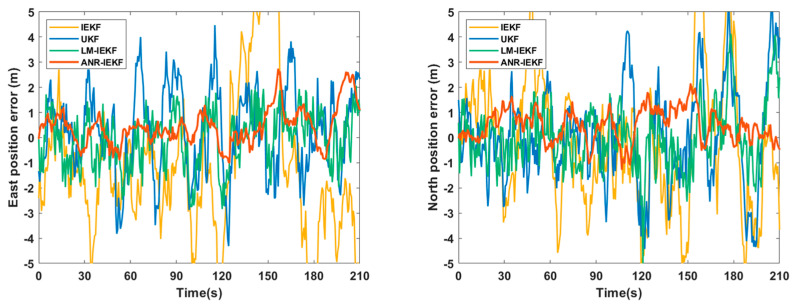
Comparison of east and north position errors for different filtering algorithms.

The comparative results of the east and north position errors, illustrated in Figure 12, highlight the substantial advantage of the ANR-IEKF algorithm in improving positioning accuracy. Throughout the experiment, the IEKF displayed the most significant error fluctuations, reflecting its vulnerability to nonlinear model errors and noise disturbances. While the UKF and LM-IEKF surpassed the IEKF in performance, their error curves continued to exhibit persistent oscillations, indicating potential for enhanced stability. In contrast, the ANR-IEKF successfully maintained errors within a minimal range throughout the entire duration of the experiment.

The performance gain of ANR-IEKF stems from its adaptive noise-regulation, which dynamically adjusts to real-time uncertainty, unlike filters with fixed noise models IEKF, UKF or LM-IEKF. This yields the lowest errors in Table 3 albeit with a minor computation increase for enhanced accuracy.

The quantitative comparison of RMSE values for velocity and position estimation derived from the four algorithms, as presented in Table 3, aligns closely with the trends observed in the previously discussed error curves, thereby confirming the superior performance of the ANR-IEKF algorithm. Specifically, the ANR-IEKF achieves the lowest values across all four error metrics, recording an east velocity error of 0.18 m/s, a north velocity error of 0.16 m, an east position error of 1.51 m, and a north position error of 1.76 m. These results are significantly lower than those obtained from the IEKF, UKF, and LM-IEKF algorithms. Notably, when compared to the second-best performing LM-IEKF, the ANR-IEKF exhibits improvements of approximately 30.7% in east velocity error, 36% in north velocity error, 23% in east position error, and 17% in north position error. This outcome clearly illustrates that the adaptive noise regulation mechanism utilized in the ANR-IEKF effectively enhances filtering accuracy, demonstrating superior robustness and stability in complex real-world scenarios.

### 4.2. Experimental Results from the Second Trajectory Segment

The second phase of the experiment (represented by the green trajectory in Figure 8) simulated two scenarios of GNSS signal outages by manually disabling the GNSS receiver. This phase aimed to evaluate the sustained positioning capability of the ANR-IEKF and TCN-BiLSTM fusion algorithm when only INS outputs were available. Given that the superiority of ANR-IEKF over other data fusion algorithms was established in the first trajectory segment, ANR-IEKF was consistently utilized as the data fusion method in this phase. Various neural network models were compared to assess the advantages of the proposed TCN-BiLSTM in predicting pseudo-GNSS signals. LSTM, BiLSTM, and CNN-BiLSTM served as baseline models for this comparison.

**Figure 13:**
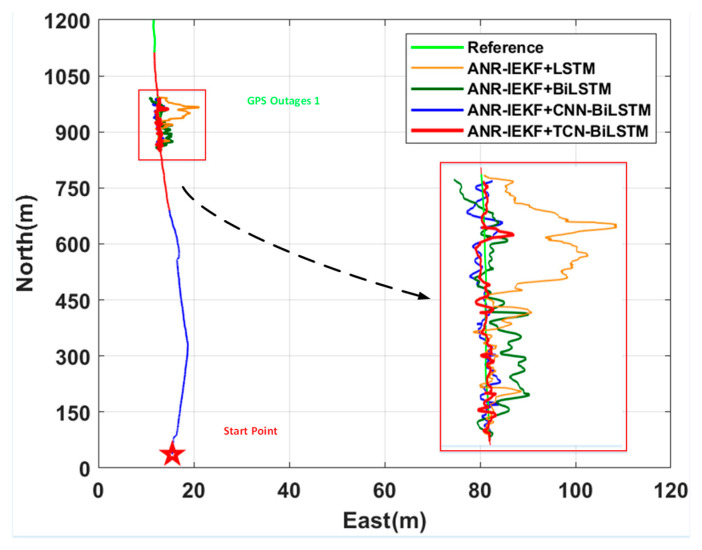
Comparison of integrated ANR-IEKF and deep learning models during GPS Outage 1.

**Figure 14:**
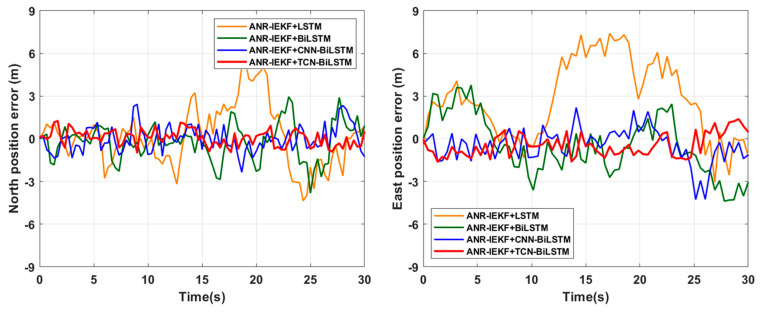
Position Error Comparison of Deep Learning Models During GPS Outage 1.

Figure 13 and Figure 14 present a comparison of the positioning results and the associated east and north errors of ANR-IEKF+TCN-BiLSTM with those of ANR-IEKF+LSTM, ANR-IEKF+BiLSTM, and ANR-IEKF+CNN-BiLSTM during the GPS Outage 1 segment. The root mean square errors (RMSEs) for the east and north positioning errors during this segment are detailed in Table 3.

To assess the efficacy of various network architectures in predicting pseudo-GNSS information during GNSS signal outages, experiments were performed on the straight-line segment identified as GPS Outage 1. The findings indicate that all four network architectures successfully generated valid pseudo-GNSS information. When integrated with INS data via the ANR-IEKF framework, these predictions yielded trajectories that aligned with the actual movement trends, thereby providing preliminary confirmation of the viability of all models for this task. However, notable performance discrepancies were observed among the different models.

The ANR-IEKF+LSTM model exhibited significant trajectory deviation in the latter segment, highlighting potential gradient decay issues inherent in standard LSTM architectures when managing long-sequence dependencies. This limitation restricts their ability to model long-term motion trends. In contrast, the ANR-IEKF+BiLSTM configuration demonstrated enhanced performance by utilizing its bidirectional encoding structure to incorporate contextual information from the trajectory, thereby facilitating more comprehensive state estimation. Nevertheless, persistent residual errors suggest that reliance on recurrent networks alone may still be inadequate for capturing the most intricate temporal patterns. Conversely, both the ANR-IEKF+CNN-BiLSTM and ANR-IEKF+TCN-BiLSTM architectures mitigate the feature extraction limitations of conventional BiLSTM by integrating specialized feature learning modules. These advanced models achieved remarkable fitting precision and stability in vehicular experiments with the generated trajectories closely aligning with the actual path.

The RMSE values of east and north errors presented in Table 3 indicate significant differences in prediction performance among the various network architectures during GNSS signal outages. Notably, the ANR-IEKF+LSTM model displays substantially higher errors in both the east and north directions compared to the other models, which can be attributed to its filtering divergence in the latter segment of the trajectory. Although the ANR-IEKF+BiLSTM model partially addresses the divergence issue, it still exhibits noticeable fluctuations in the initial segment and continues to show a divergent trend in the final segment, leading to relatively large prediction errors.

In contrast, the proposed ANR-IEKF+TCN-BiLSTM model exhibits superior trajectory prediction performance compared to all other architectures evaluated. Quantitative results indicate that for east position estimation, the model decreases the RMSE from 4.32 m (LSTM), 2.87 m (BiLSTM), and 1.74 m (CNN-BiLSTM) to 1.41 m, resulting in accuracy improvements of 67.4%, 50.9%, and 20.0%, respectively. For north position estimation, the model reduces the RMSE from 3.67 m (LSTM), 2.56 m (BiLSTM), and 1.65 m (CNN-BiLSTM) to 1.32 m, which corresponds to accuracy enhancements of 64.0%, 48.4%, and 20.0%. These findings consistently affirm the enhanced accuracy and robustness of the pseudo-GNSS signal prediction capability of the ANR-IEKF+TCN-BiLSTM in straight-line motion scenarios.

**Figure 15:**
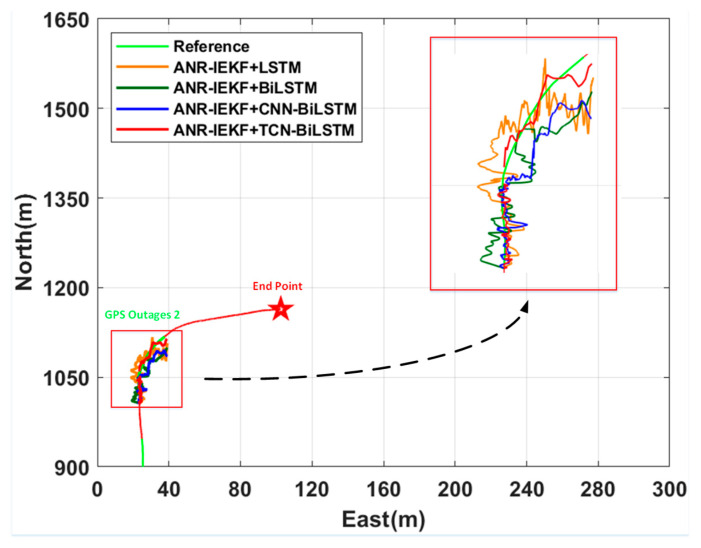
Comparison of integrated ANR-IEKF and deep learning models during GPS Outage 2.

**Figure 16:**
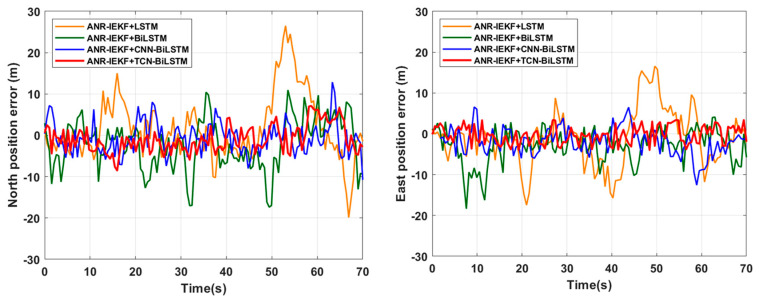
Position Error Comparison of Deep Learning Models During GPS Outage 2.

Figure 15 and Figure 16 illustrate a comparative analysis of trajectory fitting results and the associated east–west and north–south errors for the ANR-IEKF+TCN-BiLSTM framework in relation to three benchmark configurations—ANR-IEKF+LSTM, ANR-IEKF+BiLSTM, and ANR-IEKF+CNN-BiLSTM—during the GPS Outage 2 segment. Table 4 presents a quantitative evaluation of positioning accuracy, summarizing the RMSE values for both east and north position errors throughout this outage period.

In contrast to the GPS Outage 1 segment, the additional turning maneuver in the GPS Outage 2 segment places greater demands on the dynamic feature extraction and prediction capabilities of the neural networks. Within this complex motion scenario, the performance of various models shows considerable divergence. Both ANR-IEKF+LSTM and ANR-IEKF+BiLSTM exhibit significant errors during the turning phase, which are largely attributable to the constraints of their network architectures in modeling dynamic motions.

While the standard LSTM effectively captures long-term dependencies, its ability to respond to abrupt changes in vehicle motion states is limited. This limitation complicates the accurate modeling of nonlinear kinematic characteristics during turning maneuvers. Although the BiLSTM improves the overall trajectory awareness by incorporating bidirectional contextual information, its reliance on recurrent computation still renders it insufficiently sensitive to high-frequency dynamic features. This inherent constraint leads to noticeable lag and deviation at critical turning points.

The ANR-IEKF+CNN-BiLSTM model, despite demonstrating satisfactory performance in straight-line segments, shows significant errors during turning maneuvers. This limitation may arise from the CNN architecture’s primary strength in extracting local spatial features, while its ability to model implicit state transitions within continuous temporal dynamics remains constrained. Consequently, it fails to adequately capture the non-stationary characteristics of vehicle kinematics during turning processes.

In contrast, the ANR-IEKF+TCN-BiLSTM framework exhibits the smallest positioning errors throughout this segment by utilizing its distinctive temporal modeling mechanism. The TCN component effectively captures long-range dependencies through dilated convolutions and a causal structure, thereby resolving high-frequency dynamic details during steering maneuvers. When combined with the bidirectional contextual modeling capability of BiLSTM, this hybrid architecture facilitates precise feature extraction and state prediction, even in complex trajectories, thereby demonstrating superior generalization performance and robustness.

The analysis of the east and north error RMSE results presented in Table 4 demonstrates a distinct hierarchy in positioning performance among the models during GNSS outage scenarios of varying complexity. This hierarchy is a direct reflection of the inherent differences in the capacity of their network architectures for temporal modeling and feature extraction.

The stable ranking in Table 4 and Table 5 validates the TCN’s role in capturing long-term dependencies and the BiLSTM’s benefit for bidirectional refinement.

The ANR-IEKF+TCN-BiLSTM framework consistently yielded the lowest RMSE values in both the GPS Outage 1 and GPS Outage 2 segments, underscoring its remarkable robustness and generalization capabilities. This performance advantage was particularly evident in the more challenging GPS Outage 2 scenario. In terms of east position estimation, the model decreased the RMSE from 9.81 m (LSTM) and 6.10 m (BiLSTM) to 3.01 m, reflecting accuracy improvements of 69.3% and 50.7%, respectively. For north position estimation, the RMSE was reduced from 11.17 m (LSTM) and 7.43 m (BiLSTM) to 3.76 m, indicating accuracy enhancements of 66.3% and 49.4%.

In comparison to the second-best performing ANR-IEKF+CNN-BiLSTM, the proposed framework achieved further reductions in east and north errors of 37.9% and 36.4%, respectively. These findings underscore the distinctive advantage of the TCN architecture, which, through its dilated causal convolutions, effectively captures long-range dependencies and intricate dynamic features during turning maneuvers.

In comparison, the ANR-IEKF+LSTM configuration consistently exhibited the highest errors across all scenarios with its performance gap relative to the TCN-BiLSTM reaching severalfold during Outage 2. This finding highlights the inherent limitation of standard LSTM in modeling long-term dependencies during abrupt transitions in motion states. The ANR-IEKF+BiLSTM demonstrated intermediate performance; its bidirectional encoding mechanism provided moderate improvements but did not fundamentally address the recurrent neural network’s limitations in dynamic feature extraction. Meanwhile, the ANR-IEKF+CNN-BiLSTM ranked second only to the TCN-BiLSTM, suggesting that the integration of CNN modules effectively enhanced local feature extraction. However, its ability to model global temporal patterns remained slightly inferior to that of the specifically designed TCN architecture for sequential data processing.

**Figure 17:**
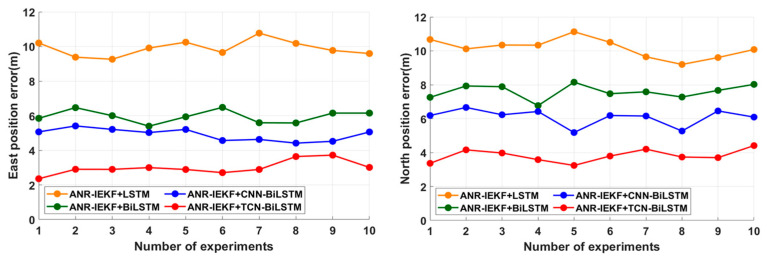
Model Consistency Across 10 Runs During GPS Outage 2.

To visually assess the consistency and stability of each model under identical configurations and similar trajectory conditions, we present the RMSE position errors obtained across all 10 independent experimental runs for the challenging GPS Outage 2 (Second Trajectory Segment) in Figure 17.

From Figure 17, it can be seen that the proposed ANR-IEKF+CNN-BiLSTM method can maintain relatively high navigation accuracy and output relatively stable results in multiple similar experiments. This also reflects its good performance under satellite denied conditions.

In summary, the RMSE data comprehensively validate that the proposed ANR-IEKF+TCN-BiLSTM model provides the most accurate and reliable pseudo-GNSS signal prediction and trajectory fitting performance in GNSS-denied environments.

### 4.3. Limitations and Future Perspectives

This paper has several limitations that outline the boundaries of its application and point to future research directions. The framework’s performance is intrinsically linked to the operational conditions reflected in its training data, including specific vehicle dynamics and sensor characteristics, which may limit its plug-and-play applicability. Furthermore, the dependence of the TCN-BiLSTM model on large volumes of high-quality training data and the inherent drift of INS during extremely long GNSS outages pose practical challenges for deployment in diverse or demanding scenarios.

These limitations naturally suggest valuable avenues for future work. Efforts could focus on developing domain-adaptive or meta-learning techniques to improve cross-platform robustness, exploring semi-supervised learning frameworks to alleviate data dependency, and investigating tightly coupled integration with complementary sensors to bound positioning errors in environments with sustained GNSS denial.

## 5. Conclusions

This paper tackles the challenge of ensuring continuous vehicle positioning during GNSS signal outages by proposing a novel integrated navigation model that combines an ANR-IEKF with a TCN-BiLSTM architecture. The effectiveness of this framework arises from the synergistic interaction between its two core components. The ANR-IEKF component provides a stable and reliable filtering foundation through its adaptive noise regulation mechanism, which effectively addresses the uncertainties inherent in neural network predictions and optimally fuses pseudo-GNSS measurements with INS data. This integration prevents filter divergence and ensures trajectory smoothness.

The TCN-BiLSTM architecture effectively combines the complementary strengths of temporal convolutional networks and bidirectional recurrent networks. The dilated convolutions of the TCN facilitate the effective capture of long-range dependencies, while the BiLSTM offers comprehensive bidirectional context modeling. This dual capability leads to minimal positioning errors across various scenarios, including both straight-line and turning maneuvers. Experimental results indicate that the integrated ANR-IEKF+TCN-BiLSTM framework achieves the highest accuracy and robustness in trajectory predictions within the specified GNSS-denied scenarios. These findings not only confirm the superior performance of the TCN-BiLSTM in pseudo-GNSS measurement prediction but also underscore the essential role of the ANR-IEKF module in dynamic noise estimation and the fusion of multi-source information.

Looking forward, this paper opens several promising avenues. Future research could focus on enhancing the model’s generalization across diverse vehicle platforms and unseen urban layouts, potentially through domain adaptation or meta-learning techniques. Additionally, integrating other sensor modalities within the same adaptive filtering framework could further bolster resilience in prolonged or extreme denial scenarios. From a practical standpoint, the proposed framework demonstrates a viable pathway toward deploying high-precision, learning-augmented navigation systems in autonomous vehicles and drones, where reliability in complex environments is paramount. Implementing the model on embedded hardware with optimization for real-time inference remains a key engineering step toward this goal.

## Figures and Tables

**Table 1:** Specifications and parameters of the equipment.

Instrument	Parameter	Value
INS	Accelerometer bias	10 mg
	Accelerometer scale factor	0.5–1%
	Gyro bias	0.5°/h
	Gyro random walk	$0.2^{{}^{\circ}}/\sqrt{h}$
GNSS	Position precision	2.5 m
	Velocity precision	0.5 m/s
	Time precision	60 ns
Reference system (NovAtel SPAN-LCI)	Position precision	1 cm ± 1 ppm
	Velocity precision	0.03 m/s
	Time precision	20 ns

**Table 2:** Configuration of the TCN-BiLSTM-ANR-IEKF model.

Module	Parameter	Value
TCN	Number of Layers	4
	Dilation Coefficients	[1,2,4,8]
	Number of Filters/Kernel Size	64/3
BiLSTM	Number of Layers	2
	Hidden State Dimension	128
Training Configuration	Optimizer	Adam
	Initial Learning Rate	1 × 10^−3^
	Batch Size	32

**Table 3:** RMSE velocity and position error values and computation time for different algorithms.

Algorithm	East Velocity Error (m/s)	North Velocity Error (m/s)	East Position Error (m)	North Position Error (m)	Computation Time (s)
IEKF	0.48	0.41	3.57	3.26	38.72
UKF	0.39	0.32	2.72	2.98	51.33
LM-IEKF	0.26	0.25	1.96	2.12	44.28
ANR-IEKF	0.18	0.16	1.51	1.76	46.57

**Table 4:** RMSE position errors of different methods during GPS Outage 1.

Outage Section	Method	East Position Error (m)	North Position Error (m)
1	ANR-IEKF+LSTM	4.32	3.67
	ANR-IEKF+BiLSTM	2.87	2.56
	ANR-IEKF+CNN-BiLSTM	1.74	1.65
	ANR-IEKF+TCN-BiLSTM	1.41	1.32

**Table 5:** RMSE position errors of different methods during GPS outages 2.

Outage Section	Method	East Position Error (m)	North Position Error (m)
2	ANR-IEKF+LSTM	9.81	11.17
	ANR-IEKF+BiLSTM	6.10	7.43
	ANR-IEKF+CNN-BiLSTM	4.85	5.91
	ANR-IEKF+TCN-BiLSTM	3.01	3.76

## Data Availability

The datasets generated during the current study are available from the corresponding author on reasonable request. The source observational data are not publicly available due to legal restrictions.
